# Genotype-phenotype correlations of *STXBP1* pathogenic variants and the treatment choices for *STXBP1*-related disorders in China

**DOI:** 10.1186/s12920-023-01474-2

**Published:** 2023-03-07

**Authors:** Miriam Kessi, Baiyu Chen, Li-Dan Shan, Ying Wang, Lifen Yang, Fei Yin, Fang He, Jing Peng, Guoli Wang

**Affiliations:** 1 Department of Pediatrics, Xiangya Hospital, Central South University, 410008 Changsha, Hunan China; 2 Hunan Intellectual and Developmental Disabilities Research Center, Changsha, Hunan China

**Keywords:** *STXBP1*-related disorders, Genotype-phenotype correlation, Intellectual disability, Global developmental delay, Epilepsy, Treatments

## Abstract

**Background:**

We aimed to analyze the genotype-phenotype correlations of *STXBP1* pathogenic variants, prognostic factors and the treatment choices in a case-series of *STXBP1*-related disorders from China.

**Methods:**

The clinical data and genetic results of the children diagnosed with *STXBP1*-related disorders at Xiangya hospital from 2011 to 2019 were collected retrospectively, and analyzed. We divided our patients into groups for comparison purposes: patients with missense variants and nonsense variants, patients who are seizure-free and not seizure-free, patients with mild to moderate intellectual disability (ID) and severe to profound global developmental delay (GDD).

**Results:**

Nineteen patients were enrolled: 17 (89.5%) unrelated and 2 (10.5%) familial. Twelve (63.2%) were females. Developmental epileptic encephalopathy (DEE) was observed in 18 (94.7%) patients and ID alone in 1 (5.3%) individual. Thirteen patients (68.4%) had profound ID/GDD, 4 (23.53%) severe, 1 (5.9%) moderate and 1 (5.9%) mild. Three patients (15.8%) with profound ID died. A total of 19 variants were detected: pathogenic (n = 15) and likely pathogenic (n = 4). Seven were novel variants: c.664-1G>-, M486R, H245N, H498Pfs*44, L41R, L410del, and D90H. Of the 8 previous reported variants, 2 were recurrent: R406C and R292C. Anti-seizure medications were used in combinations, and 7 patients became seizure-free, and most of them achieved seizure freedom within the first 2 years of life irrespective of the type of the mutation. Effective medications for the seizure-free individuals included adrenocorticotropic (ACTH) and/or levetiracetam and/or phenobarbital and/or sodium valproate and/or topiramate and/or vigabatrin and/or nitrazepam. There was no correlation between the types of pathogenic variants and the phenotypes.

**Conclusion:**

Our case-series showed that there is no genotype-phenotype correlation in patients with *STXBP1*-related disorders. This study adds 7 novel variants which expand the spectrum of *STXBP1*-related disorders. Combinations of levetiracetam and/or sodium valproate and/or ACTH and/or phenobarbital and/or vigabatrin and/or topiramate and/or nitrazepam were more often associated with seizure freedom in our cohort within 2 years of life.

**Supplementary Information:**

The online version contains supplementary material available at 10.1186/s12920-023-01474-2.

## Background

Syntaxin-binding protein 1 (*STXBP1*) is located on chromosome 9q34.3, and it encodes for an important protein that regulates neurotransmitter release [1]. *STXBP1*-related disorders are spectrum of conditions characterized by neurodevelopmental abnormalities and epilepsy [2–4]. The *STXBP1* spectrum comprises early onset epileptic encephalopathy (EOEE), neurodevelopmental disorders, developmental epileptic encephalopathy (DEE), West syndrome (WS), Ohtahara syndrome (OS), and atypical Rett syndrome [4]. The predicted incidence rate of the *STXBP1*-related disorders is 3.30–3.81 per 100 000 births [5]. A few studies from China have evaluated genotype-phenotype correlations of *STXBP1* pathogenic variants [6–8]. Some studies from other parts of the world have also examined the topic [2, 4].

This study aims to analyze the genotype-phenotype correlations of *STXBP1* pathogenic variants, prognostic factors and the treatment options. This study will expand the understanding of *STXBP1*-related disorders, clinical management and research for potential treatment.

## Materials and methods

### Ethical clearance

Written consents were obtained from the parents or guardians of the subjects which were approved by the Institutional Ethics Committee of Xiangya Hospital, Central South University, China.

### Participants

The clinical data of the patients diagnosed with *STXBP1*-related disorders and referred to Xiangya hospital, Central South University from 2011 to 2019 were collected retrospectively. We included all patients with DEE/developmental encephalopathy (DE) pathogenic/likely pathogenic variants of *STXBP1*.

### Data collection

Retrospective data was collected including demographics, onset age, medical history, seizure semiology, family history, growth and development record, physical examination (head circumference, malformations, muscle tone, other neurologic examinations), treatment provided, and treatment outcomes. Additional data that was collected included blood glucose, complete blood count, electrolytes, urea, creatinine, AST, ALT, creatine kinase, ammonia, lactate, homocysteine, serum ceruloplasmin, electroencephalograph (EEG), magnetic resonance imaging (MRI), and intelligence test (IQ/DQ).

### Routine methods of intelligence assessment

The degree of intellectual disability (ID) or global developmental delay (GDD) was assessed using the diagnostic criteria of the DSM-5 for intellectual disabilities (Diagnostic and Statistical Manual of Mental Disorders, 5th Edition, American Psychiatric Association, 2013). Standardized age-related rating scales, clinical interview and observations were used for the assessment of adaptive functioning. However, diagnosis was often made by clinical judgment instead of using formal standardized assessments, especially for young patients [9]. Standardized age-related rating scales that were used included: Gesell Developmental Schedules for patients younger than 2–4 years, Wechsler Preschool and Primary Scale of Intelligence-Fourth Edition (WPPSI-IV) for patients between 4 and 6 years, and Wechsler Intelligence Scale for Children-Fourth Edition (WISC-IV) for patients who were 6 years old or above. More details regarding assessment of intelligence can be found in our previous studies [10, 11].

### Grouping of the patients

We divided our patients into groups for comparison purposes: groups of patients with missense (including non-frameshift) and nonsense (including splice and frameshift) pathogenic variants, patients who were seizure-free and not seizure-free (including the deceased ones), and groups with mild to moderate ID/GDD and severe to profound ID/GDD (including the deceased ones).

### Evaluation of the efficacy of anti-seizure medications

Patients were followed retrospectively to determine their response towards anti-seizure medications. Seizure freedom was defined as lack of seizures for more than one year. A medicine that led to the reduction of seizures frequency by 50% within the first month of the administration was considered to be effective. The last follow up was on November 2022.

### Mutation analysis

For the children and parents who consented to genetic testing, 2 millimeters of blood were collected in EDTA anticoagulant tube, and the DNA was extracted within 72 h. Genetic tests included whole exome sequencing (WES), sub-whole exome sequencing (SWES), customized multigene panel and molecular inverted probe (MIP). Samples were tested at the precision medical genetic testing company (www.precisionmdx.com). Sanger sequencing was used to verify the parental origin of the identified variants. The genetic results were collected and interpreted according to the variant curation guidelines published in 2015 by the American College of Medical Genetics (ACMG) [12]. The pathogenicity of all detected variants was rated as: pathogenic (P), likely pathogenic (LP), variant of unknown significance (VUS), likely benign (LB) and benign (B).

### T vector sequencing

For families with possible mosaicism, at least 100 T vectors from suspected mosaicism sources were sequenced to calculate the mutation ratio.

### Statistical analysis

Statistical analysis was done using SPSS Version 25 (IBM, Armonk, NY). Categorical data was summarized in the form of frequencies and proportions. Univariate statistical analysis was carried out to compare different clinical variables (including age of onset, sex, EEG, brain imaging features, and severity of ID/GDD) among groups of the patients with missense and nonsense pathogenic variants. Similar comparison was done for patients who were seizure-free and not seizure-free, and patients with mild to moderate ID/GDD and severe to profound ID/GDD in order to identify the prognostic factors. The Chi-squared test or Fisher’s exact test was used when applicable. Results with a value of P ≤ 0.05 were considered to be statistically significant.

## Results

### Baseline characteristics of the whole cohort

A total of 19 patients were enrolled in this study, and 12 (63.2%) were females. Onset age of seizures ranged from 1 day to 1 year; and it was 3 months for 14 (73.7%) individuals. DEE was observed in 18 (94.7%) patients; whereas ID only was seen in 1 (5.2%) individual. Thirteen patients (68.4%) had profound ID/GDD, and 4 (23.53%) had severe ID/GDD (Table 1). Three children with profound GDD died: one at the age of 2 years, one at the age of 6 months and one at the age of 7 years. The causes of deaths were severe uncontrolled epilepsy for 2 patients and unknown for one child.

**Table 1:** Baseline characteristics of the whole cohort

Variable	Total number of cases (n = 19)	Percentage (%)
**Sex**		
Male	7	36.8%
Female	12	63.2%
**Ethnicity**		
Han	19	100%
Non-Han	0	0%
**Age of onset of seizures**		
≤ 3 m	14	73.7%
4–12 m	4	21.1%
> 12 m	1	5.3%
**Presence of seizures**		
Yes	18	94.7%
No	1	5.3%
**Intellectual disability/global developmental delay categories**		
Mild	1	5.9%
Moderate	1	5.9%
Severe	4	23.53%
Profound	13	68.4%
**Presence of hypsarrhythmia in an EEG**		
Yes	11	57.9%
No	7	36.8%
No EEG results	1	5.3%
**Presence of burst-suppression in EEG**		
Yes	7	36.8%
No	11	57.9%
No EEG results	1	5.3%
**Brain MRI results**		
Normal	13	68.4%
Abnormal	5	26.3%
No MRI results	1	5.3%

**Abbreviations**: EEG: electroencephalograph, m; month, MRI; magnetic resonance imaging.

### Genetic analysis results

**Fig. 1:**
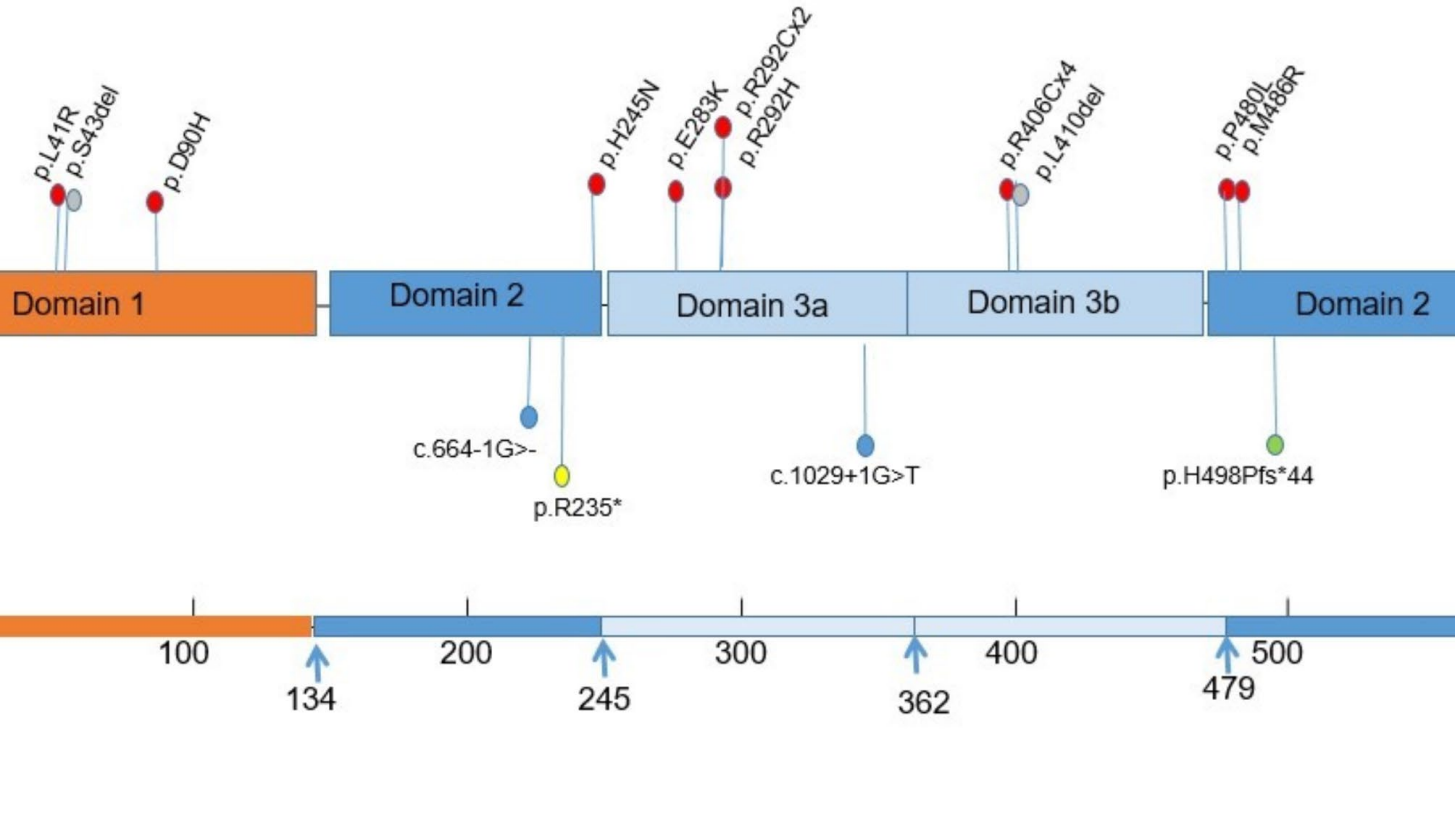
This figure shows the distributions of the identified *STXBPI* variants. Red circles represent missense pathogenic variants, grey circles represent non-frameshift pathogenic variants, blue circles represent splice pathogenic variants, yellow circles represent nonsense pathogenic variant, and green circles represent frameshift pathogenic variant

A total of 19 variants were detected in those 19 patients: pathogenic (n = 15) and likely pathogenic (n = 4). We found 15 different *STXBP1* variants: 7 novel and 8 reported in previous studies. The novel variants were c.664-1G>-, M486R, H245N, H498Pfs*44, L41R, L410del, and D90H. Of the 6 previous reported variants [4, 17–20, 22, 24–30], 2 variants were recurrent (detected in ≥ 2 children): R406C, R292C. The details of the variants have been submitted to ClinVar public database (https://www.ncbi.nlm.nih.gov/clinvar). The pathogenic variants were *de novo* for 16 individuals and inherited from maternal side for 3 children (Table 2). The mother of the two non-twin siblings (P5 and P6) had DEE and the mother of P14 had history of febrile seizures but she is well now. The P5 and P6’ mother had mild ID and had a history of experiencing syncope. Figure 1 summarizes the locations of the identified variants. Notably, maternal mosaicism was discovered in mother of P5 and P6 (Fig. 2A **and** Fig. 2B). All variants were detected by the next generation sequencing (NGS), including 11 patients of WES, 2 patients of SWES, 3 individuals of epileptic gene panel, and 2 with MIP sequencing of nerve development related genes.

**Table 2:** Genetic analysis results

Case	Genomic position	Base alteration	Amino acid change	Exon	Domain	Pathogenic variant type	Parental origin	ACMG scoring	Score	Reported/Not reported
P1	Chr9:130438188	c.1216 C > T	p.R406C	14	3b	Missense	De novo	PS1 + PS2 + PS4 + PM2	P	[24, 26, 27]
P2	Chr9:130434297	c.664-1G>-	-	9	3a	Splice	De novo	PVS1 + PS2 + PM2	P	No
P3	Chr9:130430439	c.875G > A	p.R292H	10	3a	Missense	De novo	PS1 + PS2 + PS4 + PM2	P	[28, 29]
P4	Chr9:130438188	c.1216 C > T	p.R406C	14	3b	Missense	De novo	PS1 + PS2 + PS4 + PM2	P	[24, 26, 27]
P5	Chr9:130430438	c.874 C > T	p.R292C	10	3a	Missense	Maternal	PS1 + PS4 + PM2 + PP1	P	[2]
P6	Chr9:130430438	c.874 C > T	p.R292C	10	3a	Missense	Maternal	PS1 + PS4 + PM2 + PP1	P	[2]
P7	Chr9:130440807	c.1457T > G	p.M486R	16	2	Missense	De novo	PS2 + PM2	LP	No
P8	Chr9:130438188	c.1216 C > T	p.R406C	14	3b	Missense	De novo	PS1 + PS2 + PS4 + PM2	P	[24, 26, 27]
P9	Chr9:130428514	c.733 C > A	p.H245N	8	2	Missense	De novo	PS2 + PM2 + PP2	P	No
P10	Chr9:130434396	c.1029 + 1G > T	-	12	3a	Splice	De novo	PS1 + PS2 + PM2	P	[30]
P11	Chr9:130442466	c.1493_1505del	p.H498Pfs*44	17	2	Frameshift	De novo	PVS1 + PS2 + PM2	LP	No
P12	Chr9:130440789	c.1439 C > T	p.P480L	17	2	Missense	De novo	PS1 + PS2 + PS4 + PM2	P	[4, 17–20]
P13	Chr9:130438188	c.1216 C > T	p.R406C	14	3b	Missense	De novo	PS1 + PS2 + PS4 + PM2	P	[24, 26, 27]
P14	Chr9:130430411	c.847G > A	p.E283K	10	3a	Missense	Maternal	PS1 + PM2 + PP1	P	[19]
P15	Chr9:130416028	C.122T > G	p.L41R	3	1	Missense	De novo	PS1 + PS2 + PS4 + PM2	P	No
P16	Chr9:130438197	c.1227_1229del	p.L410del	14	3b	Non frameshift	De novo	PS2 + PM2 + PM4	LP	No
P17	Chr9:130422330	c.268G > C	p.D90H	5	1	Missense	De novo	PS2 + PS4 + PM2	P	No
P18	Chr9:130416030	c.128_130del	p.S43del	3	1	Non frameshift	De novo	PS2 + PM2	LP	[22]
P19	Chr9:130428484	c.703 C > T	p.R235*	10	2	Nonsense	De novo	PVS1 + PS1 + PM2	P	[25]

**Abbreviation**s: P; pathogenic, LP: likely pathogenic.

**Fig. 2A:**
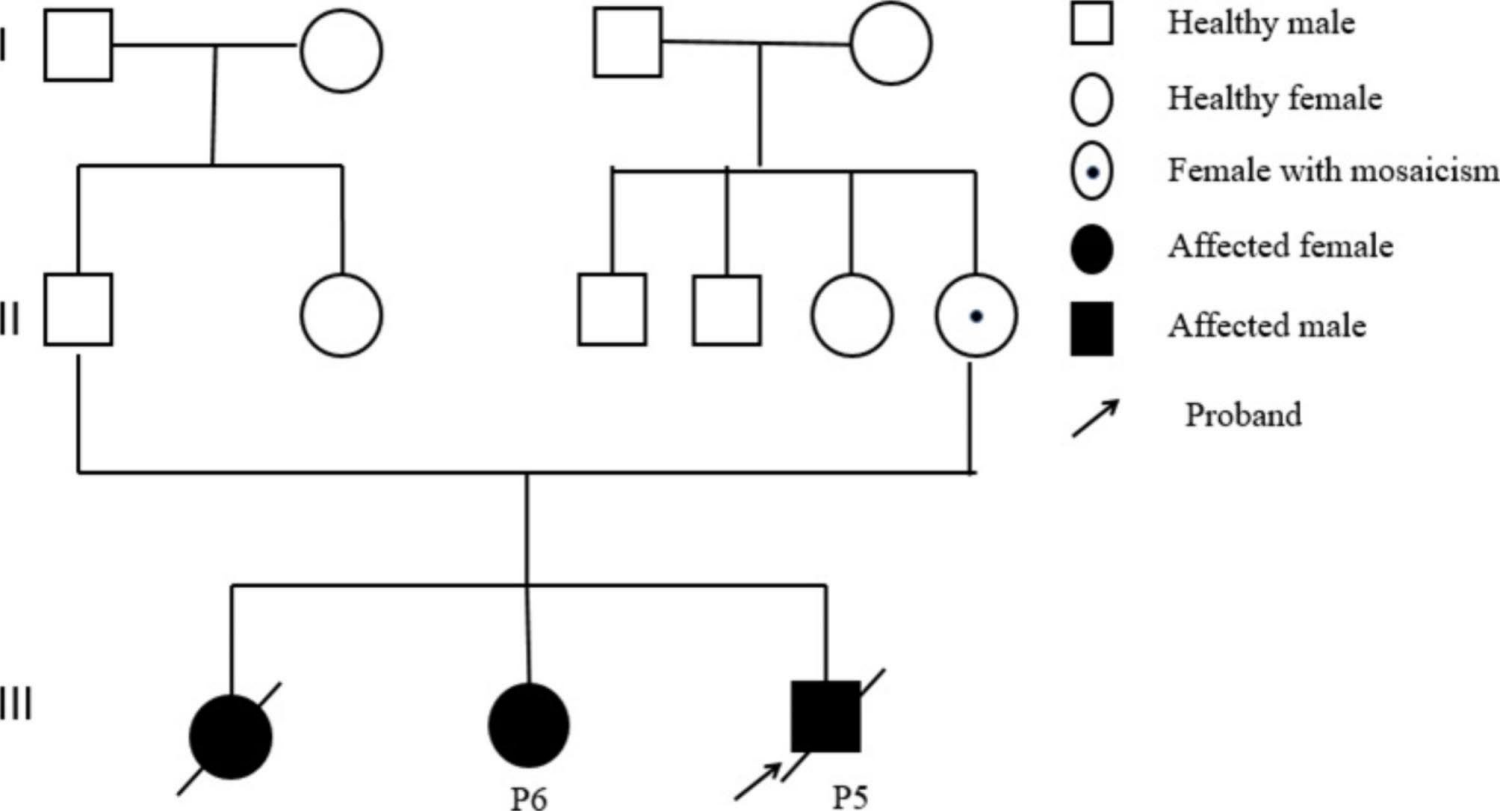
Pedigree map of patient 5 (P5) and patient 6 (P6) with p.R292C pathogenic variant. Squares represent males, circles represent females, filled squares and circles represent individuals affected, and circle with a small dot inside represent maternal mosaicism of the p.R292C pathogenic variant

**Fig. 2B:**
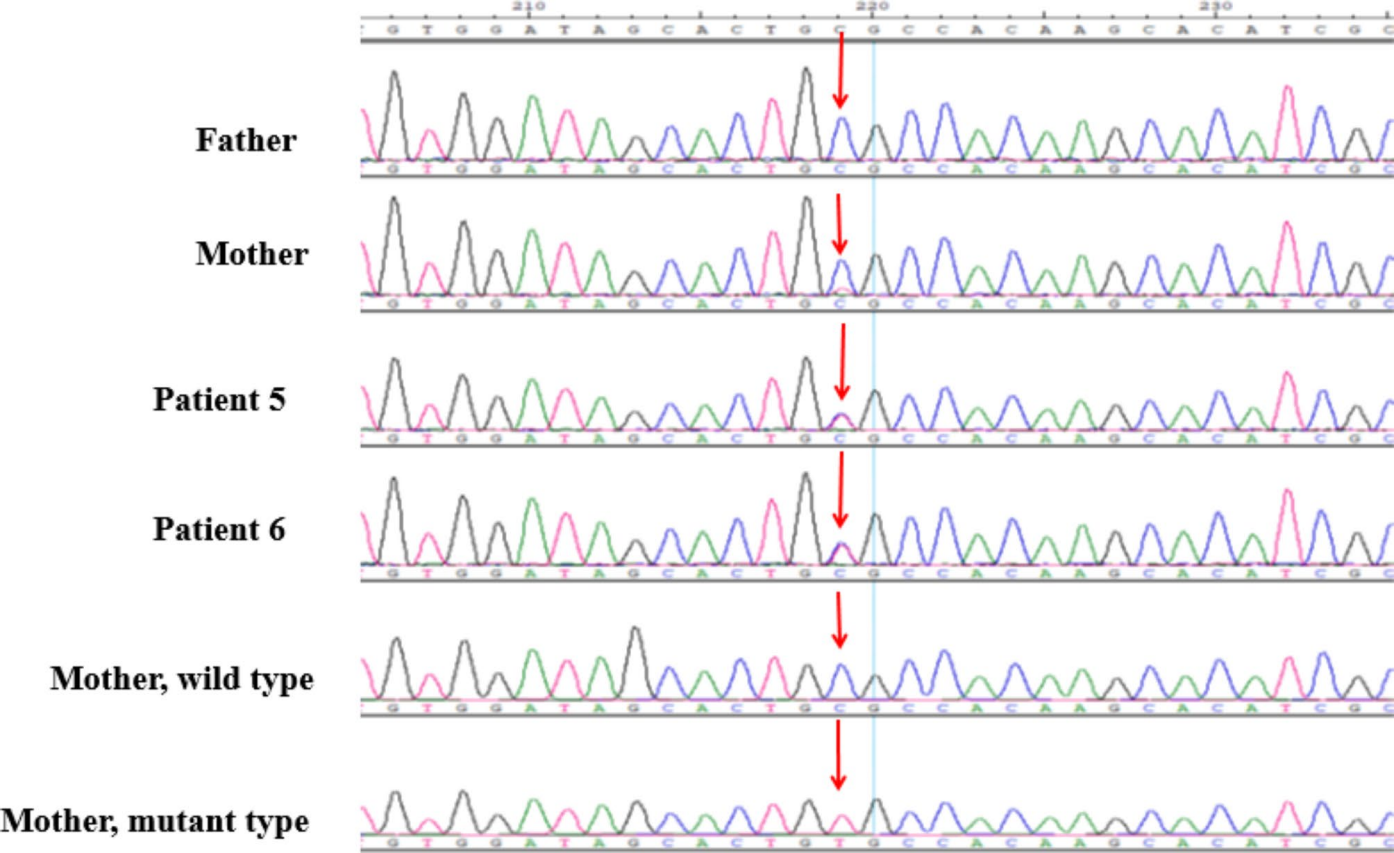
p.R292C Sanger sequencing map and mother T vector sequencing peak map. Arrows indicate pathogenic variant sites

### Phenotypes, treatment and outcomes of the patients with missense and non-frameshift pathogenic variants

Fifteen patients (78.95%) carried missense and non-frameshift pathogenic variants. The onset age ranged from 2 days to 3 months, and the mean current age is 7.7 years. Fourteen patients (93.3%) had DEE while 1 (6.7%) had ID only. Eleven patients (73.3%) had severe to profound ID/GDD. Five patients (33.3%) were hypertonic, 6 (40%) had normal muscle tone, 3 (20%) were hypotonic, and 1 (6.7%) was hypotonic while young but changed to hypertonic as she grew up. Of the 14 children with DEE, 5 (35.7%) had OS initially but later evolved to WS; 3 (21.4%) had EOEE initially but evolved to WS later; and 3 (21.4%) had WS from the beginning. The commonest initial seizure classification of those 14 individuals were as follows: 5 (35.7%) presented with focal seizures at the beginning, 1 (7.1%) with tonic-clonic, 6 (42.8%) with spasms, and 5 (35.7%) with tonic seizures. Nevertheless, the seizure semiology changed later. Five (35.7%) of 14 children with brain imaging results information had abnormal conventional brain MRI. Of those 5 patients, 1 (20%) had thin corpus callosum, 1 (20%) had arachnoid cyst on the right side, 1 (20%) had thickened parietal cortex, 1 (20%) had widened cerebral lateral ventricle mostly on the left side, and 1 (20%) had increased T1WI signal in bilateral basal ganglia, bilateral thalamus and brainstem dentate nucleus.

Out of the 15 individuals that presented with missense and non-frameshift pathogenic variants, 14 patients (93.3%) had anti-seizure medications information; and 1 patient (7.1%) who had ID was therefore exempted. Two patients (14.2%) received one anti-seizure medication, 2 patients (14.2%) received 2 anti-seizure medications, 3 patients (21.4%) received 3 anti-seizure medications 3 patients (21.4%) received 4 anti-seizure drugs, and 3 patients (21.4%) received ≥ 5 anti-seizure drugs. Of those 14 individuals, 6 (42.9%) became seizure-free, 6 (42.9%) were not seizure-free and 2 (14.2%) died. Five (83.3%) of the 6 seizure-free cases achieved seizure freedom within 2 years of life. Efficient drugs for seizure-free patients included the combination of ACTH and/or levetiracetam and/or phenobarbital and/or sodium valproate and/or topiramate and/or vigabatrin. Table 3 summarizes this information.

**Table 3:** Phenotypes, treatment and outcomes of the patients with missense and non-frameshift pathogenic variants

Patient number	Pathogenic variant type	Sex	Current age (y)	Diagnosis	Seizure onset age	Initial seizure semiology (s)	Latest seizure semiology (s)	EEG manifestations	MRI findings	Previous medicine (s)	Recent medicine (s)	Efficient medicine (s)	Seizure outcome (age)	Muscle tone	Degree of ID/GDD
P1	Missense	F	5.4	OS evolved to WS	2m10d	FS	TS, S, FMS, FCS	H/BS/M	A	TPM,VPA, ACTH and PRD	LEV	ACTH and LEV	NSF	High	Profound
P3	Missense	F	5.3	EOEE evolved to WS	16d	FS	FS, S	M	N	PB and LEV	LEV	PB and LEV	SF (0.3 y)	Normal	Severe
P4	Missense	M	11.3	WS	2 m	TCS, S	TCS, S	ESE/H/ M	A	VPA, TPM, ACTH and PRD	No medication	VPA and ACTH	SF (1 y)	High	Profound
P5	Missense	M	Died	OS evolved to WS	2d	FS, S	TS, S, FS,	H/BS	A	LEV	Died	-	Died (2 y)	High	Died
P6	Missense	F	9.8	ID, EP	7 m	TS	TS	No results	No results	No medicine	No medicine	No medicine	NSF	Low/high	Profound
P7	Missense	M	4.8	OS evolved to WS	1m19d	S	S, FS, FS-GTS	H/BS/W/F	N	LEV, TPM, ACTH, CZP, and NZP	LEV	ACTH	SF (0.6 y)	Normal	Severe
P8	Missense	M	Died	OS evolved to WS	2m28d	TS, S	S, TS	BS/F	A	VPA and ACTH	Died	None	Died (0.5 y)	Normal	Died
P9	Missense	F	9	OS evolved to WS	0.5 m	S, TS	S, FS, T	H/BS/M/W	N	TPM, ACTH, PRD, LEV, NZP, and VNS	LEV, TPM	None	NSF	High	Profound
P12	Missense	M	7.8	ID	10 m	Not applicable	Not applicable	Slow back ground	N	Not applicable	Not applicable	Not applicable	Not applicable	High	Profound
P13	Missense	F	13	ID, EP	5 m	TS	TS	W	A	LEV	No medication	LEV	SF (10y)	Normal	Moderate
P14	Missense	F	4.1	GDD, EP	1y3m	TS	TS, FS	M	N	VPA and LEV	LEV	LEV	NSF	Normal	Mild
P15	Missense	F	5.6	WS	2d	FS	FS, S	M	N	TPM and VPA	TPM, VPA	TPM and VPA	SF (2y)	Low	Severe
P16	Non frameshift	F	5.8	WS	7d	S	S, TS	H/W	N	VPA, ACTH, and VGB	VPA	ACTH and VGB	NSF	Low	Severe
P17	Missense	F	5.4	EOEE evolved to WS	1m23d	FS	FS, TS, S	W	N	LEV, TPM, and VPA	LEV and VPA	LEV and VPA	SF (2y)	Normal	Profound
P18	Non frameshift	F	5.4	EOEE evolved to WS	4 m	TS	TS, S, FS,	W/H/F	N	LEV, VPA, ACTH, KD, VGB, and CLB	VGB	LEV, VPA and VGB	NSF	Low	Profound

Abbreviations: A; abnormal, BS; burst-suppression, d; day, EOEE; early onset epileptic encephalopathy, EEG; electroencephalography, ESE; epileptic status epilepticus, F; female, FCS; focal-clonic seizures, FS; focal seizures, FS-GTCS; focal seizures secondary to generalized tonic-clonic seizures, FS-S; focal spasm seizures, AAS; atypical aphasia, GDD; global developmental delay, H; hypsarrhythmia, ID; intellectual disability, m; month, M; male; MRI; magnetic resonance imaging, M; multifocal discharge, N; normal, OS- ohtahara syndrome, S; spasms seizures, TS; tonic seizures, TCS; tonic clonic seizures, W; widespread discharge, WS; west syndrome, ACTH; adrenocorticotropic hormone, CZP; clonazepam, KD; ketogenic diet, LEV; levetiracetam, NZP; nitrazepam, PRD; prednisone tablets, PB; phenobarbital, P; patient, TPM; topiramate, VPA; sodium valproate, VGB; vigabatrin, CLB; clobazam, VNS; vagus nerve stimulation, SF; seizure free, NS; not seizure-free

### Phenotypes, treatment and outcomes of the patients with nonsense pathogenic variants

Four patients (21%) out of 19 patients had nonsense pathogenic variants. The onset age ranged from 1 day to 2 months. Two patients (50%) had OS that evolved to WS and 2 patients (50%) had EOEE that evolved to WS. Two patients (50%) presented with focal seizures at the beginning. Two patients (50%) were hypertonic, 1 (25%) was hypotonic and 1 (25%) had alternating muscle tone (high/low) as she grew up. All 4 patients (100%) had profound ID/GDD. At the end of follow up, 1 patient (25%) became seizure-free at the age of 8 years, and 2 patients (50%) were not seizure-free and 1 patient (25%) died. ACTH and levetiracetam were effective for the patient who achieved seizure freedom (Table 4).

**Table 4:** Phenotypes, treatment and outcomes of the patients with nonsense (including splice and frameshift) pathogenic variants

Patient number	Pathogenic variant type	Sex	Current age	Diagnosis	Seizure onset age	Initial seizure semiology (s)	Latest seizure semiology (s)	EEG manifestations	MRI findings	Previous drug (s)	Current drug (s)	Efficient drug (s)	Seizure outcome	Muscle tone	Degree of ID/GDD
P2	Splice	F	6.8y	OS evolved to WS	8d	FS	FS, TS, S	H/BS/W	N	ACTH, PRD, NZP, and LEV	VPA, TPM, and CBZ	ACTH and LEV	NSF	High	Profound
P10	Splice	F	9.6y	EOEE evolved to WS	2 m	FS	S, FS	H	N	ACTH, PRD, VPA, and NZP	None	ACTH and NZP	SF (8y)	High/Low	Profound
P11	Frameshift	M	Died (7y)	EOEE evolved to WS	1d	TS	TS, S, AAS	H/W/M	N	LEV, ACTH, PRD, VPA, TPM, and NZP	Died(7y)	LEV, ACTH and TPM	Died (7y4m)	High	Profound
P19	Nonsense	M	7.6y	OS evolved to WS	2 m	TS, FS	TS, FS, S	BS/H/M	N	TPM, VPA, VGB, and LEV	LEV and VPA	VPA, VGB and LEV	NSF	Low	Profound

**Abbreviations**: d; days, EP; epilepsy, EOEE; early onset epileptic encephalopathy, F; female, FS; focal seizure, GDD; global developmental delay, ID; intellectual disability, M; male, m: months, OS; ohtahara syndrome, S; spasms seizure, TS; tonic seizure, TCS; tonic clonic seizure, W: widespread spike, y; years.

### The overall seizure outcomes for both groups of patients with missense and non-frameshift pathogenic variants as well as patients with nonsense (including splice and frameshift) pathogenic variants

At the end of follow up, 7 patients (38.9%) became seizure-free and most of them achieved seizure freedom at the age of ≤ 2 years. Few individuals had early seizure onset but delayed offset. Patients whose current age ranged from 6 to 9 years, lacked seizure freedom in comparison to the younger age (< 6 years) and older age (> 9 years).

### Comparison of phenotypes between groups of patients with missense against those with nonsense pathogenic variants

The comparison between the groups of patients who carried missense against those with nonsense pathogenic variants was carried out. However, there was no statistically significant difference observed between the groups (**Supplementary Table 1**).

**Factors associated with seizure-freedom according to univariate analysis**.

The presence of epileptic spasms, onset age, hypsarrhythmia, burst suppression pattern, type of the pathogenic variant, and the utilization of ACTH, sodium valproate, levetiracetam and topiramate did not show any association with seizure outcome (**Supplementary Table 2**).

**Factors associated with the severity of the intellectual disability/global developmental delay according to univariate analysis**.

The presence or absence of burst suppression pattern, presence or absence of hypsarrhythmia, abnormal brain imaging results, seizure outcome and the type of the pathogenic variant did not show any association with the degree of severity of ID/GDD (**Supplementary Table 3**).

## Discussion

This case series encompassed 17 unrelated and 2 familial patients in which the genotypes and phenotypes of *STXBP1*-related disorders were analyzed. A total of 15 variants were detected, of which 7 were novel and 2 were recurrent. The initial clinical diagnosis included DEE and ID only. All individuals had different degrees of ID/GDD, mainly severe to profound. Majority of the patients presented with epileptic spasms and focal seizures. We could not find genotype-phenotype correlation. Some patients presented with abnormal movements. Seizure-free patients in both groups of patients with missense and nonsense pathogenic variants received the combination of ACTH and/or levetiracetam and/or phenobarbital and/or sodium valproate and/or topiramate and/or vigabatrin and/or nitrazepam.

The proportion of missense pathogenic variants was the highest in our study which is similar to the recent study [3]. A recent study has revealed that splice site, frameshift variants, whole gene deletions and partial gene deletions were linked with WS, infantile spasms, and ataxia; while patients with missense variants were more likely to have other DEE [3]. Nevertheless, our study and two other studies did not find a genotype-phenotype correlation [2, 13]. Lack of the genotype-phenotype correlations in our study can be due to the small sample size to obtain spontaneous clusters of sub-phenotypes and too heterogeneous for a holistic approach.

Our study revealed 4 patients with p.R406C variant and EOEE, WS, and DEE; which corroborates the recent findings of recent publications that genetic hotspots for STXBP1 include p.R406C (n = 40) followed by p.R292C /H/L/P (n = 30)[3]. Although our study showed that the pathogenic variant sites of P5 and P6 were the same (p.R292C), and they were siblings with the same genetic background; however, their phenotypes were different. One female sibling had similar phenotype as P5, however, the genetic results were missing. Nevertheless, their mother had mild phenotype. Notably, maternal mosaicism was discovered in the mother of P5 and P6, which is similar to previous reports [14–16]. In this study, the pathogenic variant site of P12 was p. P480L but his phenotype differ from the previous reported individuals; the phenotype of this child was a profound ID complicated with ataxia while the 2 previously reported individuals presented with WS [17–20].

All of our patients presented with different degrees of ID/GDD, mainly severe to profound, and all except one presented with DEE which is similar to the review [2]. A recent study conducted in adults suggested that severe cognitive impairments and movement disorders involving multiple systems are often present in *STXBP1*-DEE; which correlates with our findings [21]. In addition, it has been revealed that the age of seizure onset is correlated with severity of ID/GDD; a later seizure onset is associated with better developmental outcome [22]. Burst suppression pattern in EEG has been reported to relate with worse seizure prognosis [23], and it is linked with p.R406C/H variants [4]. Nevertheless, we could not establish such a relationship in our study due to a small sample size.

Most of our patients presented with seizures within the first year of life. It has been reported that seizure frequency is highest in the first year of life, but decreases dramatically by the age of 7 years [4]. Seizure freedom was achieved within 2 years of life for the majority of our patients, but we have several patients who continued to have seizures at 6–9 years. It has been reported that *STXBP-* related disorders can have prolonged seizure-free periods but never achieving permanent seizure control [21]. We have observed this pattern in three of our patients: P6, P11 and P16. Consequently, we are not certain whether the seizure freedom among the remained cases will be sustainable. It is difficult to control epileptic seizures in *STXBP1*-related disorders, and therapies are not helpful for ID/GDD. Most patients need to be treated with two or more anti-seizure medications. In this study, individuals who received the combinations of the ACTH and/or levetiracetam and/or phenobarbital and/or sodium valproate and/or topiramate and/or vigabatrin and/or nitrazepam achieved seizure freedom within the first 2 years of life which is similar to one study which reported that anti-seizure medications response were limited to the first 2 years of life [4]. Notably, ACTH and phenobarbital were also reported recently to have beneficial effect in seizure frequency reduction in WS and focal seizures, respectively [4]. In our cohort, none of the medicines seem to be superior to others. One of our patients was treated with VNS, but it aggravated seizures. There is no previous report of patients treated with VNS, indicating that further studies are required about the therapeutic effect of VNS for this condition.

## Conclusion

*STXBP1*-related disorders is a spectrum of disease whose major clinical manifestations include DEE, ID, and abnormal movements. It begins from the age of 1 month and presents with different types of seizures. Most of the patients present with WS as initial or final epileptic syndrome. It is difficult to control seizures and improve cognition. However, the combination of the ACTH and/or levetiracetam and/or phenobarbital and/or sodium valproate and/or topiramate and/or vigabatrin and/or nitrazepam seem to be effective in seizures management especially in the first 2 years of life. Some cases may have a temporary seizure freedom that continues later in life. This study adds 7 novel variants which expand the mutational spectrum of *STXBP1*-related disorders.

## Limitations

This study involves a small sample size and it was retrospective in nature. We could not find genotype-phenotype correlations since the sample size was too small to obtain spontaneous clusters of sub-phenotypes, and too heterogeneous for a holistic approach as many of our patients evolved from one condition to another. The retrospective nature of the study makes the revision of instrumental (EEG/MRI) and laboratory (e.g., genetic) investigations quite approximate. It could not provide the details of the efficacy of the individual drugs used. Future prospective studies are invited to expand the understanding of this devastating condition.

## Electronic supplementary material

Below is the link to the electronic supplementary material.

Supplementary Material 1

Supplementary Material 2

Supplementary Material 3

## Data Availability

The datasets generated and/or analysed during the current study are available in the ClinVar public database (clinvar@ncbi.nlm.nih.gov) repository.

## List of Abbreviations

ACTH: Adrenocorticotropic hormone
B: Benign
DSM-5: Diagnostic and Statistical Manual of Mental Disorders, Fifth Edition, American Psychiatric Association, 2013
EEG: Electroencephalograph
MRI: Magnetic resonance imaging
ID: Intellectual disability
GDD: Global developmental delay
DEE: Developmental epileptic encephalopathy
DE: Developmental encephalopathy
EOEE: Early onset epileptic encephalopathy
VNS: Vagus nerve stimulation
STXBP1-E: STXBP1-encephalopathy
WES: Whole exome sequencing
SWES: Sub-whole exome sequencing
MIP: Customized multigene panel and molecular inverted probe
NGS: Next generation sequencing
WS: West syndrome
OS: Ohtahara syndrome
P: Pathogenic
LP: Likely pathogenic
VUS: Variant of unknown significance
LB: Likely benign
