# Supplementary material for: Genotype-phenotype correlations of STXBP1 pathogenic variants and the treatment choices for STXBP1-related disorders in China
Source: BMC Med Genomics. 2023 Mar 7;16:46. doi: 10.1186/s12920-023-01474-2 (PMC9990233; doi:10.1186/s12920-023-01474-2)
Supplement: Supplementary file 1 — Supplementary Material 1 [file 12920_2023_1474_MOESM1_ESM.docx]

**Supplementary Table 1: Comparison of phenotypes between groups of patients with missense against those with nonsense pathogenic variants**

| **Variable** | **Group of patients with missense variants** | **Group of patients with nonsense variants** | **Total (percentage)** | **P value** |
| --- | --- | --- | --- | --- |
| **Onset age of symptoms** |  |  |  |  |
| ≤ 3 m | 10 (66.7%) | 4 (100%) | 14 (73. %) | 0.530 |
| >3 m | 5 (33.3%) | 0 (0%) | 5 (26.3%) |  |
| **Sex** |  |  |  |  |
| Male | 5 (33.3%) | 2 (50%) | 7 (36.8%) | 0.603 |
| Female | 10 (66.7%) | 2 (50%) | 12 (63.2%) |  |
| **Presence of spasms** |  |  |  |  |
| Yes | 10 (71.4%) | 4 (100%) | 14 (77.8%) | 0.524 |
| No | 4 (28.6%) | 0 (0%) | 4 (100%) |  |
| **Presence of burst suppression pattern** |  |  |  |  |
| Yes | 5 (35.7%) | 2 (50%) | 7 (38.9%) | 1.000 |
| No | 9 (64.3%) | 2 (50%) | 11 (61.1%) |  |
| **Presence of hypsarrhythmia** |  |  |  |  |
| Yes | 7 (50%) | 4 (100%) | 11 (61.1%) | 0.119 |
| No | 7 (50%) | 0 (0%) | 7 (38.9%) |  |
| **Seizure outcome** |  |  |  |  |
| Seizure-free | 6 (42.9%) | 1 (25%) | 7 (38.9%) | 0.485 |
| Non seizure-free | 8 (57.1%) | 3 (75%) | 11 (61.1%) |  |
| **Severity of intellectual disability/global developmental delay** |  |  |  |  |
| Mild and moderate | 2 (13.3%) | 0 (0%) | 2 (10.5%) | 1.000 |
| Severe and profound | 13 (86.7%) | 4 (100%) | 17 (89.5%) |  |
| **Brain MRI** |  |  |  |  |
| Normal | 9 (64.3%) | 4 (100%) | 13 (72.2%) | 0.278 |
| Abnormal | 5 (35.7%) | 0 (0%) | 5 (27.8%) |  |

**Abbreviations**; m: months.
