# Supplementary material for: Genotype-phenotype correlations of STXBP1 pathogenic variants and the treatment choices for STXBP1-related disorders in China
Source: BMC Med Genomics. 2023 Mar 7;16:46. doi: 10.1186/s12920-023-01474-2 (PMC9990233; doi:10.1186/s12920-023-01474-2)
Supplement: Supplementary file 2 — Supplementary Material 2 [file 12920_2023_1474_MOESM2_ESM.docx]

**Supplementary Table 2: Factors associated with seizure freedom according to univariate analysis**

| **Characteristics** | **Seizure-free patients** | **Non seizure-free patients** | **Total value (%)** | **P value** |
| --- | --- | --- | --- | --- |
| **Presence of spasms** |  |  |  |  |
| Yes | 6(85.7%) | 8 (72.7%) | 14 (77.8%) | 1.000 |
| No | 1 (14.3%) | 3 (27.3%) | 4 (22.2%) |  |
| **Onset age** |  |  |  |  |
| ≤ 3 m | 6 (85.7%) | 8 (72.7%) | 14 (77.8%) | 1.000 |
| > 3 m | 1 (14.3%) | 3 (27.3%) | 4 (22.2%) |  |
| EEG (BS pattern) |  |  |  |  |
| Yes | 1 (14.3%) | 6 (60.0%) | 7 (41.2%) | 0.134 |
| No | 8 (85.7%) | 4 (40.0%) | 10 (58.8%) |  |
| **EEG (hypsarrhythmia)** |  |  |  |  |
| Yes | 3 (42.9%) | 8 (80.0%) | 11 (64.7%) | 0.162 |
| No | 4 (57.1%) | 2 (20.0%) | 6 (35.3%) |  |
| **Utilization of ACTH** |  |  |  |  |
| Yes | 3 (42.9%) | 7 (70.0%) | 10 (58.8%) | 0.350 |
| No | 4 (57.1%) | 3 (30.0%) | 7 (41.2%) |  |
| **Utilization of sodium valproate** |  |  |  |  |
| Yes | 5 (71.4%) | 8 (80%) | 13 (76.5%) | 1.000 |
| No | 2 (28.6%) | 2 (20%) | 4 (23.5%) |  |
| **Utilization of levetiracetam** |  |  |  |  |
| Yes | 5 (71.4%) | 7 (70%) | 12 (70.6%) | 1.000 |
| No | 2 (28.6%) | 3 (30%) | 5 (29.4%) |  |
| **Utilization of topiramate** |  |  |  |  |
| Yes | 4 (57.1%) | 4 (40.0%) | 8 (47.1%) | 0.637 |
| No | 3 (42.9%) | 6 (60.0%) | 9 (52.9%) |  |

**Abbreviations:** ACTH; adrenocorticotropic hormone, BS; burst suppression, EEG; electroencephalograph, LEV; levetiracetam, TPM; topiramate, VPA; sodium valproate.
