# Supplementary material for: Genotype-phenotype correlations of STXBP1 pathogenic variants and the treatment choices for STXBP1-related disorders in China
Source: BMC Med Genomics. 2023 Mar 7;16:46. doi: 10.1186/s12920-023-01474-2 (PMC9990233; doi:10.1186/s12920-023-01474-2)
Supplement: Supplementary file 3 — Supplementary Material 3 [file 12920_2023_1474_MOESM3_ESM.docx]

**Supplementary Table 3: Factors associated with the degree of intellectual disability/global developmental delay**

| **Variable** | **Mild to moderate ID/GDD** | **Severe and profound ID/GDD including the dead ones** | **Total (%)** | **P value** |
| --- | --- | --- | --- | --- |
| **Onset age** |  |  |  |  |
| ≤ 3 m | **2 (50%)** | **12 (80%)** | **14 (73.7%)** | **0.272** |
| > 3 m | **2 (50%)** | **3 (20%)** | **5 (26.3%)** |  |
| **Presence of spasms** |  |  |  |  |
| Yes | 2 (50%) | 12 (85.7%) | 14 (77.8%) | 0.197 |
| No | 2 (50%) | 2 (14.3%) | 4 (22.2%) |  |
| **Presence of burst suppression pattern** |  |  |  |  |
| Yes | 2 (50%) | 5 (35.7%) | 7 (38.9%) | 1.000 |
| No | 2 (50%) | 9 (64.3%) | 11 (61.1%) |  |
| **Presence or absence of hypsarrhythmia** |  |  |  |  |
| Yes | 1 (25%) | 10 (71.4%) | 11 (61.1%) | 0.245 |
| No | 3 (75%) | 4 (28.6%) | 7 (38.9%) |  |
| **Seizure outcome** |  |  |  |  |
| Seizure free | 1 (50%) | 6 (37.5%) | 7 (38.9%) | 0.641 |
| Non seizure free | 1 (50%) | 10 (62.5%) | 11 (61.1%) |  |
| **Type of the pathogenic variants** |  |  |  |  |
| Missense | 4 (100%) | 11 (73.3%) | 15 (78.9%) | 0.530 |
| Non sense | 0 (0%) | 4 (26.7%) | 4 (21.1%) |  |
| **Brain MRI** |  |  |  |  |
| Abnormal | 1 (50%) | 4 (25%) | 5 (27.8%) | 0.490 |
| Normal | 1 (50%) | 12 (75%) | 13 (72.2%) |  |

**Abbreviations:** BS; burst suppression, EEG; electroencephalograph, GDD; global developmental delay, ID; intellectual disability, MRI: magnetic resonance imaging.
